# Infiltrating neutrophils promote renal cell carcinoma progression via VEGFa/HIF2α and estrogen receptor β signals

**DOI:** 10.18632/oncotarget.4478

**Published:** 2015-06-15

**Authors:** Wenbin Song, Chiuan-Ren Yeh, Dalin He, Yong Wang, Hongjun Xie, See-Tong Pang, Luke Sien-Shih Chang, Lei Li, Shuyuan Yeh

**Affiliations:** ^1^ George Whipple Lab for Cancer Research, Departments of Urology and Pathology, University of Rochester Medical Center, Rochester, New York, USA; ^2^ Sex Hormone Research Center, Department of Urology, The First Affiliated Hospital of Xi'an Jiaotong University, Xi'an, China

**Keywords:** tumor microenvironment, RCC, Neutrophils, ERβ, VEGFa and HIF pathways

## Abstract

Neutrophils make up a significant portion of the infiltrated immune cells found in the tumor microenvironment. Here we found more infiltrated neutrophils in human renal cell carcinoma (RCC) lesions than adjacent benign areas. *In vitro* RCC studies showed that neutrophils (HL-60N cells) infiltrated toward RCC cells and subsequently enhanced RCC cell migration and invasion. Co-culture of RCC cells with HL-60N cells up-regulated ERβ, VEGFa and HIF2α mRNA levels. ERβ signals increased RCC cell migration *via* induction of the VEGFa/HIF2α pathway. Treatment of HIF inhibitor or rapamycin, or knockdown of ERβ in RCC cells reversed HL-60N-promoted RCC migration*. In vivo* data using orthotopically xenografted RCC mouse model confirmed that infiltrated neutrophils promoted RCC migration *via* modulating the expressions of ERβ, VEGFa and HIF2α signal pathways. Together, our studies revealed that neutrophils are favorably recruited to the RCC cells to promote the RCC migration and invasion. Targeting the infiltrating RCC tumor microenvironment with anti-estrogen or rapamycin may be a potential therapy to suppress RCC progression.

## INTRODUCTION

Renal cell carcinoma (RCC) is a common urologic tumor and accounts for about 3% of all human malignancies with a steady increasing rate in recent decades [1]. The efficacy of therapy for RCC is limited as most RCC patients would soon develop resistance to conventional treatments including chemotherapy and radiotherapy [2].

RCC is considered to be an immunogenic tumor and a number of immunotherapeutic approaches have been exploited [3]. There is only partial or limited benefit obtained when selective RCC patients receive immunotherapies (high dose IL-2, IFNα+bevacizumab) or targeted therapies (tyrosine kinase inhibitors, mTOR inhibitors) [4]. The growth of RCC tumor cells is greatly influenced by the immune system [5]. RCC tissues exhibit a prominent infiltration of immune cells, consisting of T cells, natural killer (NK) cells, dendritic cells (DCs) and neutrophils. Despite profoundly infiltrated immune cells, RCC is not generally eliminated by different immune effector cells. Furthermore, immune dysfunction is found in some RCC patients, which likely contributes to tumor invasion [4]. It is of clinical interest to investigate how the tumor cells become resistant and escape from immune system control, and how the tumor associated immune cells influence the RCC development.

Evidences from epidemiological and pathological studies suggest that the neutrophil to lymphocyte ratio is an important prognostic factor based on a RCC study within a large European cohort [6]. Neutrophils are one type of the key tumor-infiltrating myeloid cells to influence tumor progression [7]. Similarly to the myeloid macrophages, neutrophils also contain a subpopulation of neutrophils named tumor-associated neutrophils (TAN) [8]. However, the potential relationship between TAN infiltration and human cancer prognosis has not been systematically discussed [9]. Epidemiological evidence has suggested that neutrophil infiltration within human cancers may be associated with a poor clinical outcome, as observed in patients with metastatic clear cell carcinomas and hepatocellular carcinoma [10].

In humans, estrogens play multiple important physiological roles. Estrogen exposure could lower the risk of heart attack, osteoporosis and breast cancer [11, 12]. But conversely, estrogen exposure could stimulate the growth of breast and uterine tissues. The bio-effects of estrogens are evident through their binding to estrogen receptors (ERs) and subsequent regulation of the transcription and activation of downstream genes. There are two major subtypes of ERs, ER alpha (ERα) and ER beta (ERβ). Distributions of ERα and ERβ vary in different tissue types [12, 13, 28, 38-40].

Our previous studies have shown that ERα is barely detectable, yet ERβ is highly expressed and could promote proliferation and invasion in RCC both *in vitro* and *in vivo*. In the present study, results from RCC cell studies suggested co-culturing neutrophils with RCC cells could increase ERβ expression and its downstream signals. Additional new data showed that a positive correlation of increased ERβ levels and neutrophil infiltrations in the tumors at later stage of RCC tissues. Here, we studied the infiltrating neutrophils roles on the RCC progression with focuses on revealing functional mechanisms and how infiltrated neutrophils alter the ERα, VEGFa and HIF2α signals in the RCC.

## RESULTS

### RCC has a better capacity than surrounding normal kidney tissues to recruit neutrophils

**Figure 1:**
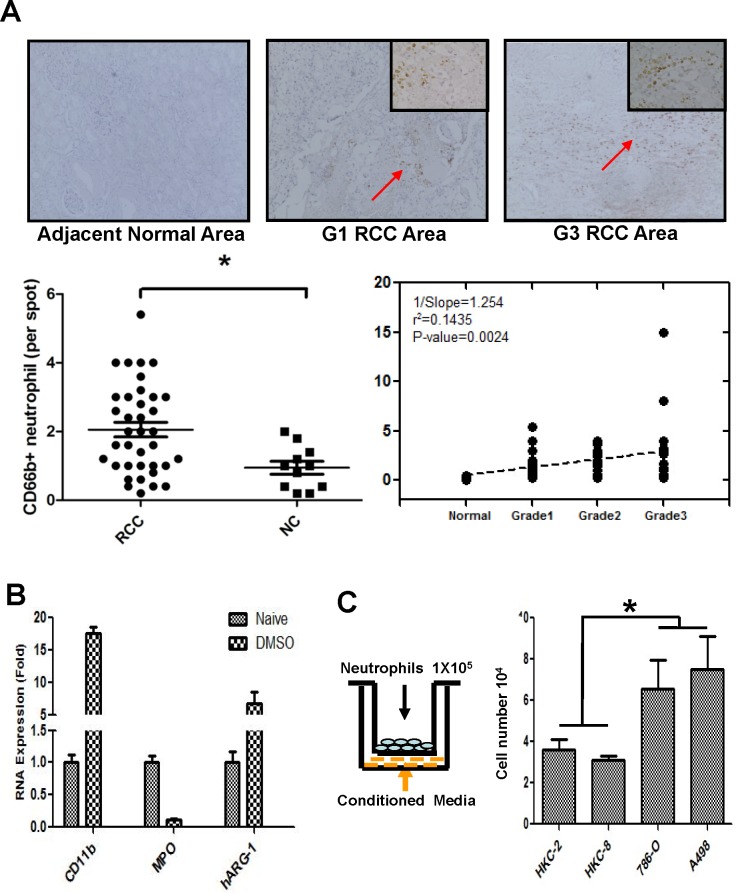
Compared to non-malignant kidney cells, RCC cells recruit more neutrophils **A.** Human clear cell RCC specimens were stained for CD66b to detect neutrophils (upper panels). IHC staining results showed that more CD66b+ signals (red arrows), which suggested more neutrophil cells infiltrations, in the renal cancer area than in the adjacent normal kidney areas. To investigate the expression of CD66b in human RCC tissues, we collected malignant tissue specimens, along with 15 adjacent normal tissues, from 60 patients who had undergone partial or radical nephrectomy for primary RCC (lower panels). All tumor tissues were diagnosed according to the 2009 edition of the TNM system and nuclear grading was performed according to WHO guidelines. The clinic-pathological parameters of these tumors are listed in Table 1. **B.** After treating with 1.25% DMSO for 5 days, HL-60 cells were differentiated to neutrophil-like cells, HL-60N. qPCR was applied to validate neutrophil marker (CD66b) and tumor associated neutrophil markers (CD11b, MPO, and hARG-1). **C.** RCC cells can better attract the HL-60N migration/infiltration. 1×10^5^ of RCC cells or non-malignant kidney epithelial cells were plated into the lower chamber of the transwell. 1×10^5^ of HL-60N cells were plated onto the upper chamber with 5 μm pore polycarbonate membrane to determine HL-60N migration rate toward conditioned media (CM) collected from RCC or non-malignant kidney cells. After 8 hrs, the HL60-N cells migrated into the lower chamber were collected and counted by the Bio-Rad TC10 automatic cell counter. Compared to the normal kidney epithelial cell lines (HKC-2 and HKC-8), RCC cells (786-O and A498) recruited more HL-60N (*P < 0.05).

To examine the potential impacts of neutrophils on RCC progression, we used IHC staining with neutrophils marker CD66b+ to compare neutrophil infiltration and results showed that more CD66b+ neutrophils were recruited to the RCC lesions than in normal kidney tissues (Figure 1A).

**Table 1:** Summery of the RCC tissues collected for the current study RCC tissue specimens were collected from 60 clear cell RCC patients who had undergone partial or radical nephrectomy for primary RCC between 2002 and 2012. All tumor tissues were evaluated according to the 2009 edition of the TNM system and nuclear grading was performed according to WHO guidelines.

Characteristics	Informative cases
Median age (range), years	56.4 (29-71)
Sex	
Male	39
Female	21
Stage	
T1	37
T2	14
T3	9
Grade	
G1	23
G2	20
G3	17
Metastasis	
M0	49
M1	11

We then applied the *in vitro* migration assay to confirm the above human clinical data. HL-60 cells were differentiated to neutrophil-like cells, HL-60N, by treating HL60 cells with 1.25% DMSO for 5 days. Tumor associated neutrophil markers, CD11b, MPO and hARG-1, were detected to validate the differentiation of neutrophils (HL-60N) (Figure 1B). To test whether RCC cells have a better capability than the non-malignant kidney cells to attract neutrophils, we applied a transwell Boyden chamber migration system. HL-60N cells were placed on the top wells, conditioned media (CM) from RCC or non-malignant kidney cells were added in the bottom wells (Figure 1C). After 8 hours of incubation, the number of HL-60N cells that migrated through the membranes were counted. Compared to the non-malignant kidney cells, HKC-2 or HKC-8, the RCC cells, 786-O and A498, have a much better capacity to recruit the HL-60N cells (Figure 1C).

Together, results from Figure 1A-1C suggest that RCC cells/tissues have a better capacity to recruit neutrophils than the surrounding normal kidney cells.

### Infiltrated neutrophils to RCC could enhance the RCC cell migration/invasion

**Figure 2:**
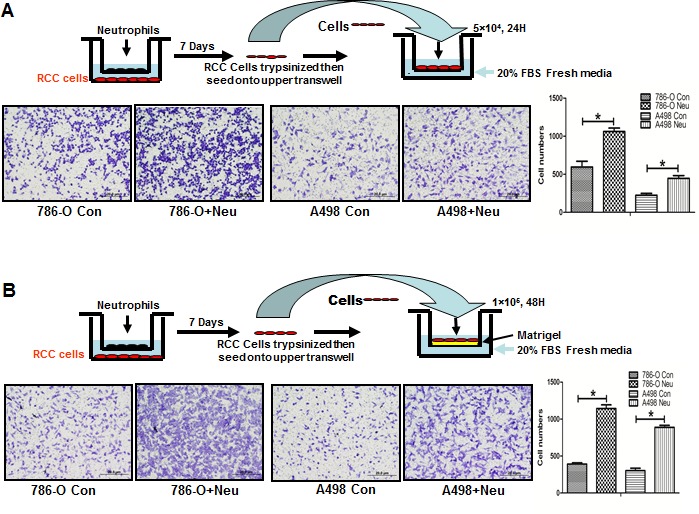
Co-culture with neutrophils promoted RCC invasion **A.** The cartoon shows the procedure to co-culture RCC and HL-60N cells and to test HL-60N cells promoted RCC migration. The RCC and HL-60N cells were co-cultured in 0.4 μm pore size transwell plates for 7 days. After co-culturing with HL-60N, RCC cells were re-seeded into the upper chamber of a new transwell with 20% FBS fresh media in the bottom chamber. The transwell migration results showed that HL-60N co-cultured RCC cells have a higher migration capability (*P < 0.05). **B.** HL-60N co-cultured RCC cells have a higher invasion capability. Using a protocol similar to A, we found that there was a higher invasion ability (through matrigel) in RCC cells after co-culture with HL-60N cells than non-co-cultured RCC cells.

To further study the consequences of infiltrated neutrophils on RCC progression (Figure 2A), we then applied transwell plates to test the migration/invasion of RCC cells with or without co-culturing with neutrophils HL-60N cells for 7 days. RCC cells were then re-seeded in the upper transwell (5×10^4^/well). The migration results showed the higher ability of migration in neutrophil-co-cultured RCC cells than non-co-cultured RCC cells (Figure 2A). In addition, the transwell invasion assay results showed that co-culture of infiltrated HL-60N cells would allow RCC 786-O cells to gain a better invasion capacity (Figure 2B, *P < 0.05). Similar results were obtained when we replaced RCC 786-O cells with the A498 cells, another RCC cell line.

### Mechanism studies: Infiltrated neutrophils could up-regulate ERβ, VEGFa and HIF2α signal pathways in RCC

**Figure 3:**
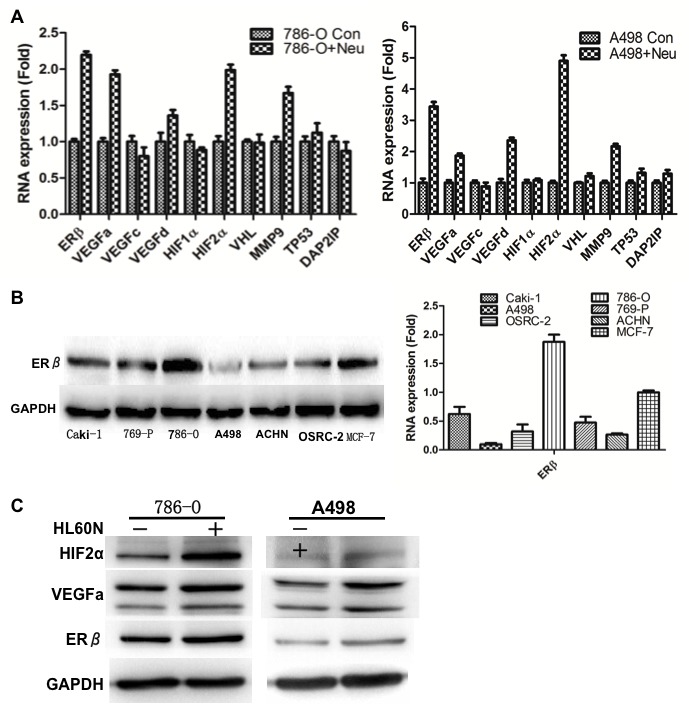
Mechanism dissection **A.** Screening gene profile changes in neutrophil co-incubated RCC cells. Q-PCR results showed mRNA expressions of VEGFa and HIF2α, but not VEGFc, VEGFd and HIF1α, were selectively and significantly increased in RCC cells after co-culture with HL-60N cells for 7 days. The data were validated in 2 different RCC cell, 786-O and A498. **B.** Detection of ERβ levels in different RCC cells. Western blot detection of ERβ in different RCC cell lines. Breast cancer cells, MCF-7, were used as a positive control for ERβ expression. ERβ has the highest expression level in 786-O and the lowest in A498 cells. qPCR was applied to check ERβ expression in RCC cell lines. **C.** Western blot detection of ERβ, VEGFa and HIF2a protein expressions after co-culture with HL-60N cells. Data showed that co-culture of HL-60N and RCC cells could increase ERβ and other invasion/angiogenesis related genes in RCC cells. GAPDH was used as control to show the equal loading of protein.

To further dissect the molecular mechanism(s) by which RCC cell invasion is enhanced after co-culture with neutrophil HL-60N cells, we applied Q-PCR-based focus-array analyses to search for the key metastasis-related genes that are responsible for ERβ-enhanced RCC progression. Among many increased metastasis-related genes, we found the expression of HIF2α and vascular endothelial growth factor a (VEGFa) (VEGFa) and ERβ expressions were selectively increased in RCC 786-O and A498 cells after co-culture with neutrophils (Figure 3A). Western blot analysis results showed ERβ expression levels were different in various RCC cells (Figure 3B). Among these RCC cells, we choose 786-O cells that have high endogenous ERβ expression and A498 cells that have relatively low ERβ expression for further functional study. Figure 3C showed the increased HIF2α, VEGFa and ERβ protein expressions in both RCC cell after co-culture with HL-60N cells.

Together, results from Figures 2-3 using different RCC cell lines demonstrated that recruited neutrophils could enhance the RCC cell migration/invasion and infiltrated neutrophils may promote RCC cells invasion *via* up-regulation of ERβ signals in RCC cells.

### Knockdown of ERβ, and treatment of HIF inhibitor or rapamycin can inhibit neutrophils-promoted RCC invasion

**Figure 4:**
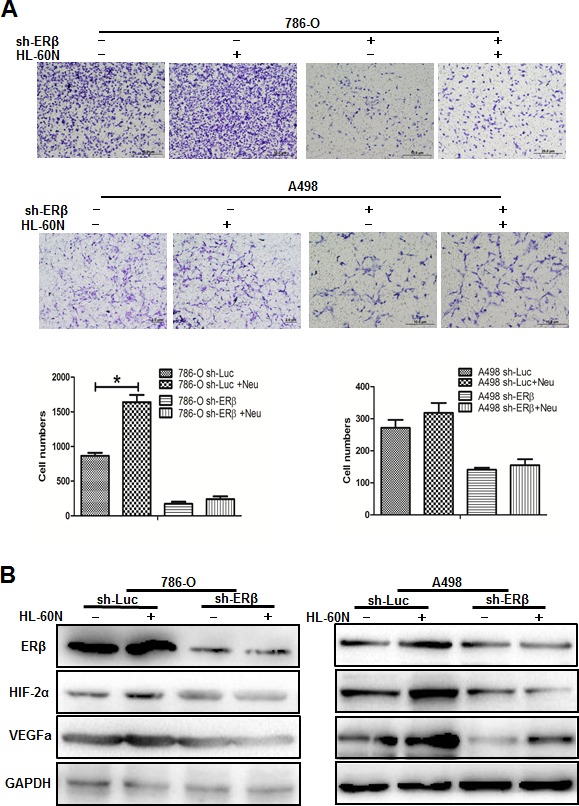
Down-regulated ERβ could regulate the down-stream VEGFa and HIF2α pathways in RCC cells **A.** We used the lentiviral-shRNA system to knock down ERβ in 786-O (with high endogenous ERβ) and A498 (with low endogenous ERβ), then co-cultured with HL-60N for 7 days. Data showed that knockdown of ERβ expression can reverse HL-60N promoted RCC invasion. **B.** Western blot data showed that ERβ, VEGFa and HIF2α protein increases in RCC cells after co-culture with HL-60N cells. Knockdown of ERβ in RCC could diminish the HL-60N co-culture promoted VEGFa and HIF2α expressions in RCC cells.

**Figure 5:**
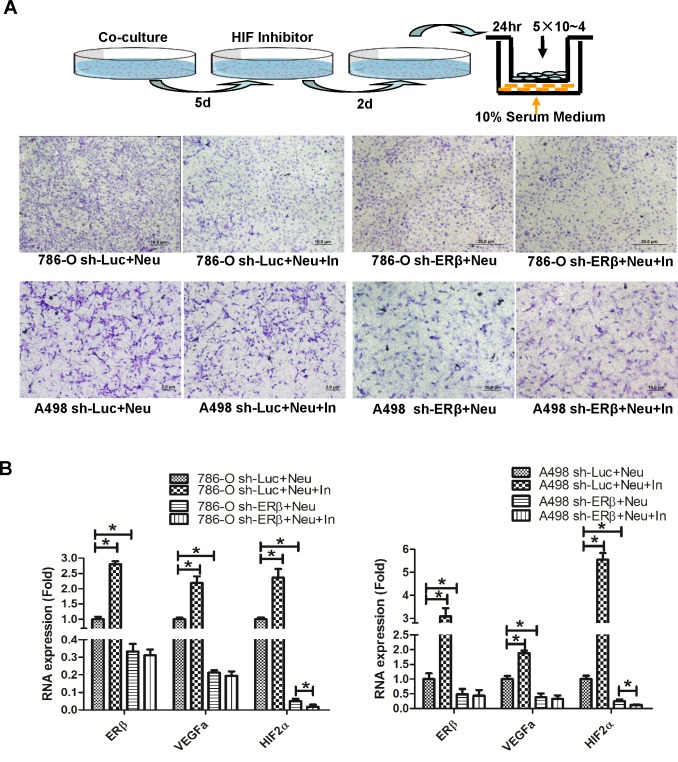

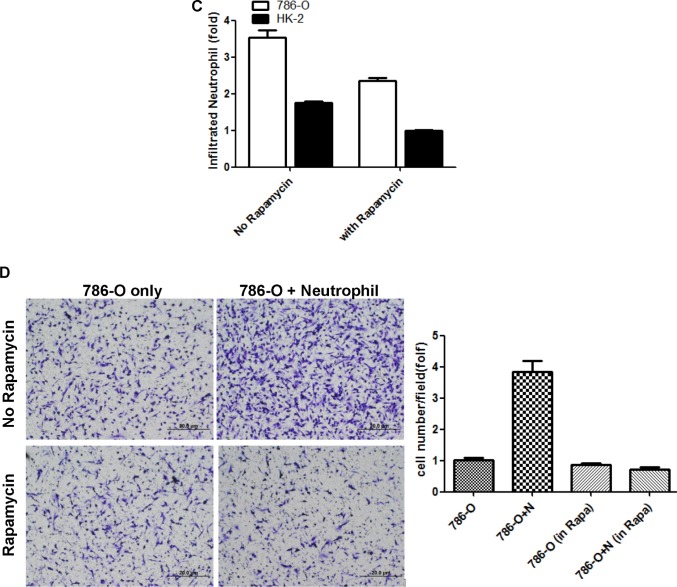
HIF inhibitor and rapamycin treatment can attenuate neutrophils-promoted RCC migration and invasion **A.** Using lentiviral system to knockdown ERβ in A498 and 786-O, those RCC cells were then co-cultured with HL-60N. After 5 days co-culture, 10 μM HIF inhibitor was added for 2 more days. RCC cells were then trypsinized and seeded in the upper chambers of transwells (5×10^4^/well) with 10% FBS media for 24 hr to determine the migration rate. The cartoon illustrates the procedure to perform the experiments. **B.** qPCR to detect ERβ, VEGFa and HIF2α expression with or without treatment of HIF inhibitor. Together, our data support that a lower ERβ expression can down-regulate VEGFa/HIF2α expression. HIF inhibitor treatment can diminish neutrophils/ERβ-stimulated RCC migration. **C.** Rapamycin treatment suppresses neutrophil infiltration. CMs collected from 786-O and HK2 cells with/without rapamycin (10ng/ml) treatment were put in bottom wells of 24 wells cell culture plate. Neutrophils were seeded into upper-transwell (pore size: 5μm). Infiltrated neutrophils were collected in bottom wells after 3 hrs of incubation. **D.** Rapamycin treatment could attenuate neutrophil-promoted RCC invasion. In the upper left panel: 786-O alone with DMSO mock treatment; the upper right panel: HL-60N co-cultured with 786-O cells with DMSO mock treatment. In the bottom left panel, 786-O cells with rapamycin treatment; in bottom right panel, HL-60N and 786-O co-culture with rapamycin treatment (10 ng/ml). RCC invasion quantification results were averaged from counting six representative fields under microscope of each condition. Results were normalized to 786-O only with DMSO treatment.

To validate the importance of ERβ, VEGFa and HIF2α in neutrophils promoted RCC invasion, we used lentiviral-ERβ lentiviral-ERβ cDNA or shRNA transduced RCC cells. We first knocked down ER in 786-O cells that have high endogenous ER expression. RCC cells were then co-incubated with neutrophils for 7 days and seeded for invasion assay. Our data showed that knockdown of ERβ in RCC cells could inhibit neutrophils-promoted RCC invasion. Interestingly and importantly, when we knocked down ERβ, we observed a reduced expression of the VEGFa and HIF2α in HL-60N co-cultured RCC cells (Figure 4). Furthermore, an interruption approach using HIF inhibitor can effectively reverse neutrophil-co-culture induced HIF2a expression and invasion in RCC cells (Figure 5A and 5B).

In addition to knocking down ERβ and treatment with HIF inhibitor, we also examined the effects of rapamycin, which was reported to inhibit the migration of neutrophils [14], or macrophages [15]. Also, Rapamycin has the potential to be used as treatment of different types of cancers [16, 17]. We tested whether rapamycin could interrupt the infiltration of neutrophils toward RCC cells as well as the neutrophils-enhanced RCC invasion. Results showed that rapamycin treatment could effectively inhibit the capability for neutrophils to infiltrate through coated transwell-membrane (Figure 5C) and consequently inhibit the neutrophils-enhanced RCC invasion (Figure 5D).

Together, results from Figures 4 and 5 suggest that infiltrated neutrophils may function through modulation of ERβ/VEGFa/HIF2α signals to enhance the RCC cell invasion. The inhibition of neutrophils by rapamycin, or by blocking ERβ and HIF2α may be applied as alternative therapy strategies to control RCC invasion.

### Infiltrated neutrophils enhanced RCC invasion in the *in vivo* mouse model

To confirm the above *in vitro* RCC cell study results in the *in vivo* pre-clinical RCC model, we then orthotopically implanted RCC 786-O and/or HL-60N cells (9:1 ratio) under the renal capsule to test the tumor growth and metastasis.

**Figure 6:**
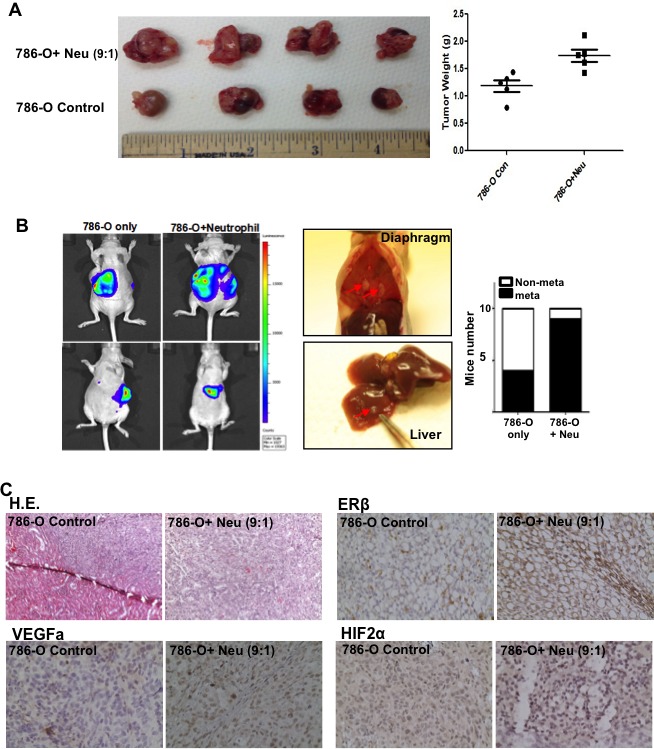

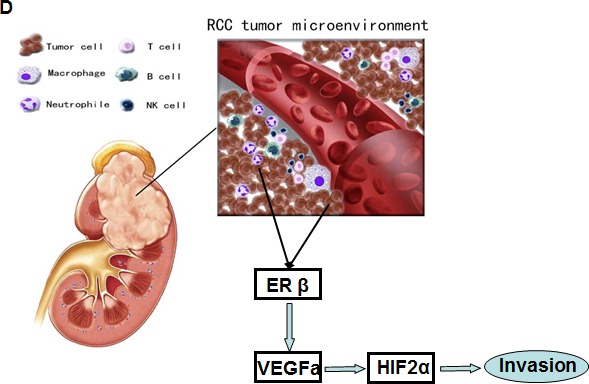
Tumor associated neutrophils could promote RCC invasion in orthotopically implanted RCC model 786-O cells (9×10^5^) and HL-60N cells (1×10^5^) were orthotopically co-implanted into the renal capsule of nude mice. After 6 weeks, the RCC growth and metastasis were evaluated. **A.** The 786-O cells co-injected with HL-60N cells showed a higher tumor growth rate. Tumor weights in each group were measured. **B.** Metastases were found in the diaphragm (middle panel, representative picture) of neutrophils co-implanted RCC tumors. RCC cells were stably transfected with luciferase cDNA in order to perform non-invasive *in vivo* imaging (IVIS), which was applied to monitor the RCC growth and metastasis. Mouse numbers with or without metastatic tumors of each group were quantitated (right panel, *n* = 10 for each group). **C.** Histology analyses of RCC tumors formed in nude mice. IHC and H&E results indicated that HL-60N co-implanted RCC tumors are positively correlated with higher ERβ (upper right panel), VEGFa (lower left panel) and HIF2α (lower right panel). Anti-ERβ antibody can detect predominate nuclei staining signals of ERβ in RCC tumors. **D.** Summary of mechanisms and regulatory pathways of neutrophils-promoted RCC progression. RCC cells can better recruit neutrophil infiltration. RCC cells and neutrophils interact with each other in the RCC tumor microenvironment. The infiltrated neutrophils can function *via* increasing ERβ to up-regulate VEGFa and HIF2α signaling and lead to an increased RCC invasion.

RCC 786-O cells were first stably transfected with luciferase and IVIS imaging was applied to monitor RCC tumor growth and metastasis 3 weeks after tumor implantation, followed by weekly IVIS detection for an additional 5 weeks. Eight weeks after tumor implantation, the mice were sacrificed for tumor characterization. Results consistently showed that tumors were bigger in the mice co-implanted with 786-O cells and HL-60N cells (Figure 6A). Representative IVIS images were shown on the left panel of Figure 6B. Metastases were found in the diaphragm of neutrophils co-implanted RCC tumors. There are higher metastatic rates in 786-O+HL-60N co-implanted mouse tumor group (9/10, 90%) as compared to 786-O cell only group (4/10, 40%) (Figure 6B). In addition, IHC staining of mouse RCC tumors showed the expressions of ERβ, VEGFa, and HIF2α markers were consistently higher in 786-O+HL-60N tumor group than 786-O tumor group (Figure 6C).

Together, results from *in vivo* mouse model studies (Figure 6A-6C) confirmed the above *in vitro* cell lines studies (Figures 3 and 4) and demonstrated that infiltrated neutrophils could enhance RCC growth and invasion *via* modulating ERβ and VEGFa/HIF2α signals.

## DISCUSSION

Besides tumor cells, the tumor microenvironment is composed of a wide spectrum of immune cell types, which can significantly affect cancer progression and patient outcome. Tumor-infiltrating neutrophils (TIN) are known to make up a significant part of the immune cells within the tumor microenvironment in different types of human cancer [18-20]. Despite their origin in the peripheral blood, TINs have been shown to exhibit impaired bactericidal and enhanced angiogenic activities [21]. The presence of intra-tumoral neutrophils has been reported to be associated with poor prognosis in primary breast cancer [22] and RCC [23]. Consistently, studies, have shown neutrophil depletion experiments led to an inhibited tumor growth [24], limited metastasis numbers [25] and reduced endothelial recruitment to the tumors [26]. Our results confirm the rather anti-tumor role of neutrophils as we found significantly higher ratios of these cells in dissociated tumor cell suspension from advanced RCC patients with T3-T4 and metastatic disease. In our study, results showed that RCC patients have higher proportions of neutrophils in tumor samples than in adjacent normal tissues. Higher percentages of neutrophil cells were found in the tumor tissues in the higher grade tumor tissues from RCC patients (Figure 1). These findings may reflect the possibility of cancer-induced systemic as well as local immunosuppression, which both seem to be the early event in the course of the disease.

In this study, we sought to determine the role of neutrophils on RCC progression and whether ERβ plays differential functions between immune cells and RCC cells. The crosstalk between immune cells and tumor cells that leads to phenotypic alterations in tumor biology has been broadly termed immunosculpting or immunoediting [27]. In this study, we sought to determine the role of neutrophils on RCC progression and whether ERβ/VEGFa/HIF2α play important roles for the interactions between immune cells and RCC cells. In addition, we found that ERβ can promote RCC progression via TGF-β/SMAD3 pathway (Song and Yeh, et al, 2015 paper submitted). Furthermore, a prior study in prostate cancer supportively showed that ligand-bound ERβ promotes the epithelial-mesenchymal transition *via* the VEGF pathway [28].

The VEGF pathway is an important regulator of angiogenesis [29]. Activation of this pathway triggers a network of signaling processes that promote endothelial cell growth, migration, and survival from preexisting vascular beds. The VEGF/VEGF-receptor axis plays an important role in the mobilization of endothelial progenitor cells from the bone marrow to distant sites of neovascularization. The well-established role of VEGF in promoting tumor angiogenesis and growth has led to the development of various biologic agents that target this pathway [29, 30]. RCC may be used as a model for the development of effective antiangiogenic therapies. It is well known that the estrogen receptors are nuclear proteins and several reports have shown the dominant nuclear staining (>95%) of ERβ. Recently, there was a report by Yu CP *et al*. showing ERβ expression is reduced in higher grade RCC and may play an inhibiting role for RCC development [31]. The reasons behind the different conclusions regarding the ERβ roles in RCC progression remain unclear. We obtained RCC cells from the ATCC bank and used those cells within 30 passages for data collection. There have been concerns regarding the histology of the ERβ signal detected by Yu CP *et al*. using rabbit anti-ERβ (Epitomics), which did not show a dominant nuclear staining [31]. Also, the specificity of this rabbit anti-ERβ (Epitomics) is not well characterized for ERβ IHC staining in the nuclear receptor field.

Neutrophils are thought to play an important role in normal physiological angiogenesis. During the menstrual cycle, angiogenesis occurs in order to support the proliferation and growth of the endometrial tissue. The source of the potent pro-angiogenic factor VEGFa in these tissues was found to be from neutrophils. In fact, neutrophils expressing VEGFa could be found in the microvessels of the endometrium during the proliferative stage of the cycle when the endometrium can quadruple in thickness [32], which suggest the potential impacts of neutrophil secreted VEGFa in tumor progression. In the present study, our data showed that ERβ increased VEGFa activity and promoted angiogenesis-related gene expressions. The detailed mechanisms by which ERβ regulates VEGFa and HIF-2α could be further delineated in future studies.

## MATERIALS AND METHODS

### Human samples and IHC staining

To investigate the expression of CD66b+ neutrophils in human RCC tissues, we collected malignant tissue specimens, as well as 15 adjacent normal tissues, from 60 clear cell RCC patients who had undergone partial or radical nephrectomy for primary RCC, at the Department of Urology, the First Affiliated Hospital of Medicine School, Xi'an Jiaotong University, between 2002 and 2012. All tumor tissues were evaluated according to the 2009 edition of the TNM system and nuclear grading was performed according to WHO guidelines.

Formalin-fixed, paraffin-embedded samples were cut to a thickness of 5 μm. Each tissue section was deparaffinized and rehydrated with graded ethanol. For antigen retrieval, the slides were put in 1 mM EDTA solution (pH 8.0) and boiled in a microwave oven for 15 min. Endogenous peroxidase activity was blocked with a 0.3% hydrogen peroxide solution for 10 min at room temperature. After rinsing with PBS, slides were incubated overnight at 4°C with respective primary antibodies which include mouse anti-human CD66b monoclonal antibody (ThermoFisher; dilution 1:50). After three washes in PBS, sections were incubated with biotinylated anti-mouse secondary antibody for 30 min at room temperature. Immunostaining was performed using the Envision System with diaminobenzidine (DakoCytomation, Glostrup, Denmark). Finally, the signal was developed with 3,3′-diaminobenzidine tetrahydrochloride (DAB), and all of the slides were counterstained with hematoxylin. Data were obtained by manually counting positively stained cells in five separate areas of intratumoral regions under 400× high-power magnification. Densities were determined by computing the mean number of positively stained cells per high power microscopic field [33, 34].

### Cell culture and stable cell lines

The human RCC cell lines 786-O (with high endogenous ERβ level), and A498 (with low endogenous ERβ) were purchased from ATCC and grown in DMEM media containing 1% antibiotics and 10% fetal bovine serum (FBS) and maintained in 10% heat-inactivated FBS RPMI media with 1% Pen/Strep. Normal human kidney proximal tubular cell lines, HKC-2/HKC-8 (ERβ positive) were kindly provided by Dr. Syed Khundmiri from University of Louisville (Louisville, KY), and maintained in Dulbucco's Minimum Essential Medium (DMEM) (Invitrogen, Carlsbad, CA) with 10% FBS. The ERβ knockdown of 786-O and ERβ overexpressing A498 cells were established by lentiviral transduction of siRNA ERβ or ERβ cDNA, respectively.

### Lentiviral expression plasmid construction and virus production

ERβ (PLKO.1-puro-shERβ) was constructed with target sequence 5′-GCGAGTAACAAGGGCATGGAA-3′ according to Addgene's pLKO.1 protocol. Lentiviral particles were generated by calcium phosphate transfection of lentiviral expressing, packaging and envelop plasmids into HEK 293 cells. Lentiviral particles were collected to infect target cells according to previous reports [35].

The 786-O and A498 cell lines were stimulated with 1.25% DMSO for 2 weeks to be differentiated into neutrophils as HL-60N. All cell lines were cultured in a 5% (v/v) CO2 humidified incubator at 37°C.

### Cell migration assay

The CM from RCC cells and normal human kidney proximal tubular cell lines, HKC-2 and HKC-8, at 1×10^5^ were plated into the lower chambers of the transwells with 5 μm pore polycarbonate membrane inserts. 1×10^5^ HL-60N cells were plated onto the upper chambers. After 8 hrs, the cells migrated into the lower chambers were collected and counted by the Bio-Rad TC10 automatic cell counter. Each data point was performed in triplicate and the experiments were independently repeated twice.

The migration capability of RCC cells was determined using the transwell assay. RCC cells were co-cultured with HL-60N for 7 days, then trypsinized and seeded with serum-free DMEM into the upper chambers at 5×10^4^ cells/well, the bottom chambers contained DMEM with 20% FBS, and incubated for 24 hr. The migrated RCC cells attached to the lower surface of the membrane were fixed by 4% paraformaldehyde and stained with 1% toluidine blue. Cell numbers were counted in five randomly chosen microscopic fields per membrane.

### Invasion assay

For *in vitro* invasion assays [36], the upper chambers of the transwells (8 μm pore size) were pre-coated with the growth factor-reduced matrigel (matrigel: serum free RPMI = 1:4) (BD Biosciences). Before invasion assays. RCC cells were co-cultured with HL-60N for 72 hrs in 6-well transwell plates (0.4 μm pore size). 1×10^4^ of HL-60N cells were plated onto the upper chambers and 1×10^5^ RCC cells were plated into the lower chambers. The conditioned media (CM) were collected, diluted with 10% FBS DMEM media at 1:1 ratio, added into the lower chambers, and the parental RCC cells (1×10^5^) with or without indicated treatment were plated onto upper chambers. After 24 hrs of incubation, the cells in the upper chamber were removed. The insert membranes were fixed in ice cold 75% alcohol, stained with crystal violet, and the positively stained cells were counted. The invaded RCC cell numbers were averaged from counting numbers of five random fields. Each data point was run in triplicate and each set of experiment was performed in triplicate.

### Quantitative PCR

Total RNA was extracted from each cell line using Trizol (Invitrogen). Reverse transcription was performed using the iScript reverse transcription kit (Bio-Rad). Quantitative real-time PCR (qRT-PCR) was conducted using a Bio-Rad CFX96 system with SYBR green to determine the levels of mRNA expression of listed genes. Expression levels were normalized to the expression of GAPDH mRNA.

### Western blot assay

Cells were washed twice in PBS and lysed with RIPA buffer containing 1% protease inhibitors (Amresco, Cochran, USA). Protein concentration in the cell lystate solution was determined by BCA protein assay (Amresco, Cochran, USA). Each cell lystate was mixed with 5×SDS-PAGE loading buffer (Amresco). Equivalent protein quantities were loaded to 7%-15% SDS-polyacrylamide gels (Bio-Rad). Proteins were electotransferred to PVDF membranes (Millipore, Atlanta GA, USA) that were blocked in Tris-buffered saline plus 0.05% Tween-20 (TBS-T) containing 5% non-fat dried milk for 1 hr. The membranes were washed in TBS-T and incubated with each primary monoclonal antibody overnight at 4°C [37]. The following primary antibodies were used: Rabbit anti-ERβ polyclonal antibody (GTX 110607, GeneTex), rabbit anti-VEGF polyclonal antibody (Abcam ab46154, 1μg/ml) and mouse anti-HIF2α monoclonal antibody (Abcam ab8365, 1:200 diluted) and mouse anti-GAPDH monoclonal antibody (Santa Cruz) were used at 1:1000 dilution. The immuno-positive bands were visualized with an ECL chemiluminescent detection system (Thermo Scientific), and the images were transferred to the Bio-rad imaging system. All analyses were performed at least in duplicate.

### Rapamycin treatment on neutrophil infiltration toward RCC cells and neutrophils-increased RCC invasion

786-O (or HK2 cells) were seeded in 6-cm cell culture dishes (1×10^5^/dish). After 24 hrs, media were refreshed and mock control or rapamycin (10 ng/ml) was added. After 48 hrs, CMs were collected for the following neutrophil recruitment assays. The CM collected from HK2 (or 786-O cells) with/without rapamycin treatment were passed through 0.45 μM filters. CMs were then added in the bottom wells of 24-well transwells. Neutrophils were seeded into the upper wells of transwells with serum free media (1×10^5^ in 150 μl media/well, pore size: 5 μm). After 3 hrs of incubation, infiltrated neutrophils in the media of the bottom wells were collected and counted.

For *in vitro* invasion assays, the upper chambers of the transwells (Corning; pore size: 8 μm) were pre-coated with matrigel containing reduced-growth factors (1:4 serum free RPMI media) (BD Biosciences, Sparks, MD). Before the invasion assays, RCC cells were cultured alone or co-cultured with HL-60N for 48 hrs in 6-well transwell plates (Corning; pore size: 0.4 μm) with/without rapamycin (10 ng/ml) treatment. 1×10^5^ of HL-60N cells were plated onto the upper chambers and 1×10^6^ RCC cells were seeded into the lower chambers. We tested 5 types of rapamycin treatments, (i) HL-60N cells were treated with rapamycin for 24 hrs, then cells were rinsed with new media twice to wash out the excess rapamycin, then co-cultured with 786-O; (ii) HL-60N and 786-O cells were both treated with rapamycin for 24 hrs, then rapamycin was washed out, and then cells remained co-cultured together; (iii) only 786-O cells were treated with rapamycin before co-culture, and (iv) HL-60N and 786-O cells were co-cultured together in the presence of rapamycin for 48 hrs. The CM were collected, diluted with 10% FBS DMEM media at the ratio of 1:1, plated into the lower chambers, and the parental RCC cells (1×10^5^) without treatment were plated onto upper chambers of transwell plates. After 18 hrs incubation, the non-invaded cells in the upper chambers were removed. The insert membranes were fixed in ice cold 75% alcohol, stained with crystal violet, and the positively stained cells attached to the bottom of the membranes were counted under the microscope. The numbers of invaded cells were averaged from counting of five random fields. Each sample was run in triplicate and in at least 3 independent experiments.

### *In vivo* metastasis studies

Female nude mice (7-8 weeks old) were purchased from NCI. Group 1 mice were implanted with 1×10^6^ 786-O cells (mixed with Matrigel, 1:1v/v) and Group 2 mice were co-implanted with 1×10^6^ 786-O cells and 1×10^5^ HL-60N cells into renal capsule, (*n* = 5/each group). At the end of experiments, the tumors grown in the renal capsule were harvested, measured, and fixed for further histopathological analysis. Metastatic foci were counted and collected [36].

### IHC for animal model

The mouse RCC tumor samples were fixed in 4% neutral buffered para-formaldehyde, and embedded in paraffin. The primary antibodies of the mouse anti-human CD66b monoclonal antibody (Thermo Fisher), mouse monoclonal antibody to ERβ (Abcam 14C8 1:50), rabbit anti-VEGF polyclonal antibody (Abcam ab46154, 1:1000) and mouse anti-HIF-2α monoclonal antibody (Abcam ab8365, 1:1500 diluted) were used for staining. The primary antibody was recognized by the biotinylated secondary antibody (Vector), and visualized by VECTASTAIN ABC peroxidase system and peroxidase substrate DAB kit (Vector) [38].

### Statistical analysis

Data are presented as mean± SD from at least 3 independent experiments. Statistical analyses involved paired t-test with SPSS 17.0 (SPSS Inc., Chicago, IL). For *in vivo* studies, measurements of tumor metastasis among the three groups were analyzed through one-way ANOVA coupled with the Newman-Keuls test. *P < 0.05* was considered statistically significant.
